# Transcriptomic characterization of cancer-testis antigens identifies *MAGEA3* as a driver of tumor progression in hepatocellular carcinoma

**DOI:** 10.1371/journal.pgen.1009589

**Published:** 2021-06-24

**Authors:** Amanda J. Craig, Teresa Garcia-Lezana, Marina Ruiz de Galarreta, Carlos Villacorta-Martin, Edgar G. Kozlova, Sebastiao N. Martins-Filho, Johann von Felden, Mehmet Eren Ahsen, Erin Bresnahan, Gabriela Hernandez-Meza, Ismail Labgaa, Delia D’Avola, Myron Schwartz, Josep M. Llovet, Daniela Sia, Swan Thung, Bojan Losic, Amaia Lujambio, Augusto Villanueva

**Affiliations:** 1 Division of Liver Diseases, Department of Medicine, Liver Cancer Program, Tisch Cancer Institute, Graduate School of Biomedical Sciences, Icahn School of Medicine at Mount Sinai, New York City, New York, United States of America; 2 Department of Oncological Sciences, The Tisch Cancer Institute, Graduate School of Biomedical Sciences, Icahn School of Medicine at Mount Sinai, New York City, New York, United States of America; 3 Precision Immunology Institute at Icahn School of Medicine at Mount Sinai, New York City, New York, United States of America; 4 Department of Genetics and Genomic Sciences, Cancer Immunology Program, Tisch Cancer Institute, Icahn School of Medicine at Mount Sinai, New York City, New York, United States of America; 5 Icahn Institute for Data Science and Genomic Technology, Icahn School of Medicine at Mount Sinai, New York City, New York, United States of America; 6 Department of Laboratory Medicine and Pathobiology, University Health Network, University of Toronto, Toronto, Canada; 7 Department of Medicine, University Medical Center Hamburg-Eppendorf, Hamburg, Germany; 8 Department of Visceral Surgery, Lausanne University Hospital CHUV, Lausanne, Switzerland; 9 Liver Unit and Centro de Investigación Biomédica en Red de Enfermedades Hepáticas y Digestivas (CIBERehd), Clínica Universidad de Navarra, Pamplona, Spain; 10 Department of Surgery, Icahn School of Medicine at Mount Sinai, New York City, New York, United States of America; 11 Translational Research Laboratory, BCLC Group, IDIBAPS, Hospital Clinic, Universitat de Barcelona, Catalonia and Madrid, Spain; 12 Institució Catalana de Recerca i Estudis Avançats, Barcelona, Catalonia, Spain; 13 Department of Pathology, Icahn School of Medicine at Mount Sinai, New York City, New York, United States of America; 14 Diabetes, Obesity and Metabolism Institute, Icahn School of Medicine at Mount Sinai, New York City, New York, United States of America; 15 Division of Hematology and Medical Oncology, Department of Medicine, Icahn School of Medicine at Mount Sinai, New York City, New York, United States of America; Amgen, Inc., UNITED STATES

## Abstract

Cancer testis antigens (CTAs) are an extensive gene family with a unique expression pattern restricted to germ cells, but aberrantly reactivated in cancer tissues. Studies indicate that the expression (or re-expression) of CTAs within the MAGE-A family is common in hepatocellular carcinoma (HCC). However, no systematic characterization has yet been reported. The aim of this study is to perform a comprehensive profile of CTA de-regulation in HCC and experimentally evaluate the role of MAGEA3 as a driver of HCC progression. The transcriptomic analysis of 44 multi-regionally sampled HCCs from 12 patients identified high intra-tumor heterogeneity of CTAs. In addition, a subset of CTAs was significantly overexpressed in histologically poorly differentiated regions. Further analysis of CTAs in larger patient cohorts revealed high CTA expression related to worse overall survival and several other markers of poor prognosis. Functional analysis of MAGEA3 was performed in human HCC cell lines by gene silencing and in a genetic mouse model by overexpression of MAGEA3 in the liver. Knockdown of MAGEA3 decreased cell proliferation, colony formation and increased apoptosis. MAGEA3 overexpression was associated with more aggressive tumors *in vivo*. In conclusion MAGEA3 enhances tumor progression and should be considered as a novel therapeutic target in HCC.

## Author summary

Hepatocellular carcinoma (HCC) is one of the leading causes of cancer related death worldwide. Several recurrent driver genes of HCC have been established, yet no targeted drug against these drivers has been approved for the treatment of HCC. Novel targets for therapeutic development are desperately needed. This study aims to identify novel drivers of tumor progression from low-grade tumors to high-grade, aggressive malignancies. We collected multiple biopsies of single tumors from 12 patients and performed RNA-sequencing. We hypothesized that we could identify important genes to HCC progression by identifying differentially up-regulated genes in high-grade regions compared to low-grade regions of the same tumor. Interestingly, we found that one family of genes was recurrently over-expressed in the most aggressive regions of the tumors: cancer testis antigens (CTAs). Here we show that X-chromosome located CTAs, and especially MAGEA3, are associated with markers of poor prognosis in HCC. Experimentally, inhibiting expression of MAGEA3 in HCC cell lines decreases the ability for cells to proliferate and causes cells to undergo cell death. In mice, over-expressing MAGEA3 in the liver along with known HCC drivers causes mice to succumb to disease faster. Changes in RNA and protein levels within HCC cells after MAGEA3 inhibition suggests that maintenance of Survivin, an apoptosis inhibiting protein, plays an important role in how MAGEA3 drives HCC progression. Overall, our study identified the CTA MAGEA3 as important for HCC progression and a novel therapeutic target.

## Introduction

Hepatocellular carcinoma (HCC), the most common form of primary liver cancer, is the fourth leading cause of cancer related mortality worldwide [1]. The incidence of HCC has been steadily increasing in the majority of Western countries over the last 25 years [2]. FDA-approved systemic therapies only prolong survival in the range of months and prognosis for advanced stage disease remains dismal. The lack of a more robust response to systemic therapies may be due to the heterogeneous nature of HCC. Molecular differences in HCC between patients are extensive, leading to different molecular classifications which can be separated into two major groups, the Proliferation and Non-proliferation classes, determined by unsupervised clustering of gene expression data [3]. More recently, several studies have described clonal evolution in HCC using multi-regional next generation sequencing [4,5]. The most commonly identified driver mutations in HCC (*TERT* promoter, *TP53* and *CTNNB1)* are notoriously hard to therapeutically target [3].

Cancer testis antigens (CTA) are a group of proteins that were initially uncovered in studies aimed to discover tumor specific antigens [6,7]. Under normal circumstances, though, a majority of CTAs are expressed mainly in male germ cells within the testes, an immune-privileged site. While CTAs have been detected in stem cells, detection is rare in somatic cells and thus these proteins are capable of eliciting an adaptive immune response [7]. Approximately half of all CTAs are located on the X chromosome and are classified as XCTAs. CTAs are believed to play a role in spermatogenesis, including the MAGE-A family, which are transiently expressed at distinct stages of meiosis and spermiogenesis [8]. Other functions of CTAs, especially the MAGE-A family, are continuing to be discovered and are broadly involved in spermatogenesis, transcriptional control, and protection against stressors which induce apoptosis [9,10]. Aberrant expression of CTAs has been observed in several tumor types, including breast, non-small-cell lung carcinoma and melanoma [10].

Expression of CTAs has been documented in HCC [11–14]. Several groups have proposed the utility of CTAs as prognostic biomarkers. Individually, MAGEA1, MAGEA3, MAGEA4, MAGEC2 and NY-ESO-1 are highly expressed in HCC and associated with metastasis, tumor recurrence, high AFP levels and poor clinical outcomes [15–17]. Currently, there is no comprehensive analysis of CTA deregulation in HCC. Studies that do exist are each individually focused on a small subset of CTA genes. This makes it difficult to give accurate estimates of the frequency with which CTAs collectively are aberrantly expressed in HCC. Additionally, previous studies have utilized cohorts of HCC patients with small sample sizes to profile CTA RNA expression and there is no consensus regarding high expression frequency across studies. Here, we present complete and exhaustive CTA expression profiling across 2 large HCC patient cohorts for the first time.

Historically, CTAs have been investigated as targets of immunotherapy due to their selective expression by tumor cells [10]. Several studies of the MAGE-A CTA family have described its role in the degradation of tumor suppressors [18,19]. All MAGE-A CTAs are located on the Xq28 locus and are highly homologous. In the context of cancer, MAGE-A genes are mainly described as contributing to apoptosis evasion [20]. *MAGEA3* is a proven tumor maintenance gene in lung, breast and colon cancer, where cell line viability was dependent on *MAGEA3* expression and overexpression was capable of transforming human colonic epithelial cells [21]. Mechanistically, MAGEA3 can regulate the E3 ubiquitin ligase TRIM28, causing the increased degradation of the tumor suppressors TP53 and AMPK [21,22]. In the context of multiple myeloma, MAGEA3 can inhibit apoptosis through repression of TP53-dependent up-regulation of BAX and both TP53-dependent and independent maintenance of Survivin expression [23]. Additionally, it is important to note that these studies among others show that MAGEA3 has a significant impact on transcriptional activity [24]. Aside from the repression of the key transcriptional regulator TP53, MAGEA3 also modulates the KRAB domain containing zinc finger transcription factors (KZNF). Depending on the type of KRAB domain present, MAGEA3 can release transcriptional repression induced by KZNFs [25,26].

In this work we leverage intra-tumoral expression differences between low and high tumor grade regions of the same tumor nodule to identify novel drivers of HCC progression on a patient-by-patient basis. To identify intra-tumoral differences, we performed RNA sequencing on tissue samples from multiple regions of the same tumor nodule in 12 patients, termed here as multi-regional RNA-seq. Using differential gene expression, we identified X chromosome located CTAs (XCTAs) as a heterogeneously expressed group of genes in HCC, especially MAGEA3, which was associated with poor prognostic markers in two large patient cohorts. Experimental evidence *in vitro* and *in vivo* identifies the role of the XCTA MAGEA3 in tumor progression through apoptosis inhibition by Survivin upregulation, which underscores the role of MAGEA3 as a novel therapeutic target in HCC.

## Results

### Regional comparison of gene expression identifies up-regulation of cancer testis antigens in poor grade areas of heterogeneous tumors

**Fig 1:**
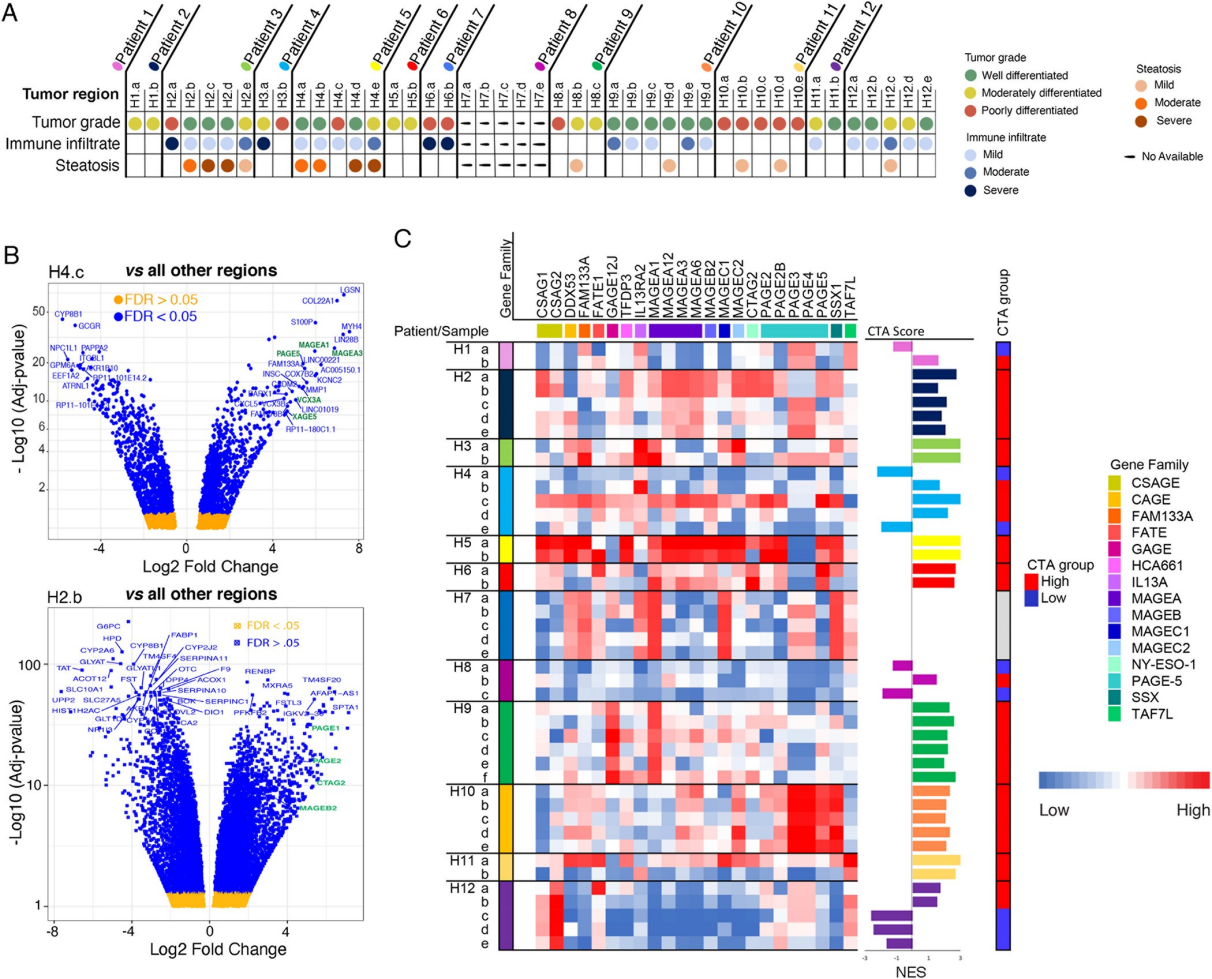
**A)** Summary of the histological characteristics (tumor grade, immune infiltrate and steatosis) of the different HCC regions analyzed in each patient of the multi-regionally sampled cohort. **B)** Representative volcano plots showing differentially expressed genes among different regions of the same tumor in two patients. The comparison was performed between the region presenting the highest histological differentiation grade (poorly differentiated) and the rest of the regions. CTA family members (green) appear highly expressed in poorly differentiated regions. Gene expression levels are represented as Log Fold Change. **C)** Gene expression profile of X chromosome located CTA genes in the multi-regionally sampled cohort. To compare expression levels, CTA enrichment scores were calculated in each region according to the CTA profile (bars). A dichotomized classification of sample CTA expression is displayed as the CTA group column (red = high, blue = low). *CTA*, *Cancer Testis Antigens; HCC*, *hepatocellular carcinoma*.

We have recently reported on the intra-tumoral dynamics between the microenvironment and tumoral cells driving tumor evolution utilizing multi-regional RNA-seq data [27]. We further believe that our multi-regionally sampled cohort is ideal to discover novel drivers of tumor progression in the context of intra-tumor heterogeneity. This is because our cohort contained several patients with variation in tumor grade between the multiple regions sampled from a single tumor nodule. Tumor samples were histologically graded using Edmondson criteria, which reflects how abnormal tumor cells appear histologically. Low grade, or well-differentiated, tumor cells still retain characteristics of normal cells, whereas high grade, or poorly differentiated, cells have a more abnormal structure and appearance. We hypothesize that tumor cells acquire alterations that cause the progression from low grade to an aggressive high grade state. By comparing intra-tumoral low and high grade areas, these progression events can be identified on a per patient basis. Our cohort consists of 44 multi-regional samples from 12 HCC patients (median of 4 regions per patient, **Fig 1A).** First, we identified genes expressed only in high-grade areas (i.e., moderate or poor vs well differentiation) of heterogeneous tumors that may play an active role in making these tumor regions more aggressive (**S1 Fig**). To identify these regional differences in gene expression, we compared differentially expressed genes in each region of the tumor of our multi-regionally sampled cohort on a patient-by-patient basis. Known HCC drivers such as *LIN28B* [28] and candidate driver genes, including a sub-set of CTAs (i.e., *MAGEA3*, *MAGEA1*), were significantly upregulated (>10 FC, FDR<0.001) in high grade regions compared to low-grade regions of the same tumor nodule (**Fig 1B**). For example, CTAs including *MAGEA3*, *MAGEA1*, *PAGE5*, *TPTE*, *VCX*, *XAGE5* and *VCX3B* were significantly upregulated in region H4.c (i.e., poorly differentiated, **S1 Fig**) compared to the other regions of patient 4. We also found heterogeneous expression of *PAGE1*, *PAGE2*, *CTAG2* and *MAGE2B* in patient 2, with expression most highly detected in region H2.a (poorly differentiated region, **Fig 1B and S1 and S2 Tables**). Comparing the expression of CTAs located in chromosome X (XCTAs) (**S3 Table**) across the sampled regions, all patients expressed at least one XCTA (**Fig 1C**). To compare CTA expression between regions of the same patient, we performed ssGSEA for those CTAs with expression limited to testis and placenta, immunoprivileged sites, with the expectation that in the context of cancer, these CTAs would be expected to produce the strongest immune response (n = 68) [6,29]. Enriched CTA scores confirmed the highly variable intratumoral expression of CTAs (**Fig 1C and S4 Table)**. Additionally, we saw no correlation between CTA expression and tumor purity or the presence of tumor infiltrating lymphocytes (TILs, previously published [27]). These data confirm significant intratumoral heterogeneous expression of CTAs in HCC.

### XCTA expression is associated with an aggressive phenotype and poor prognosis in HCC

**Fig 2:**
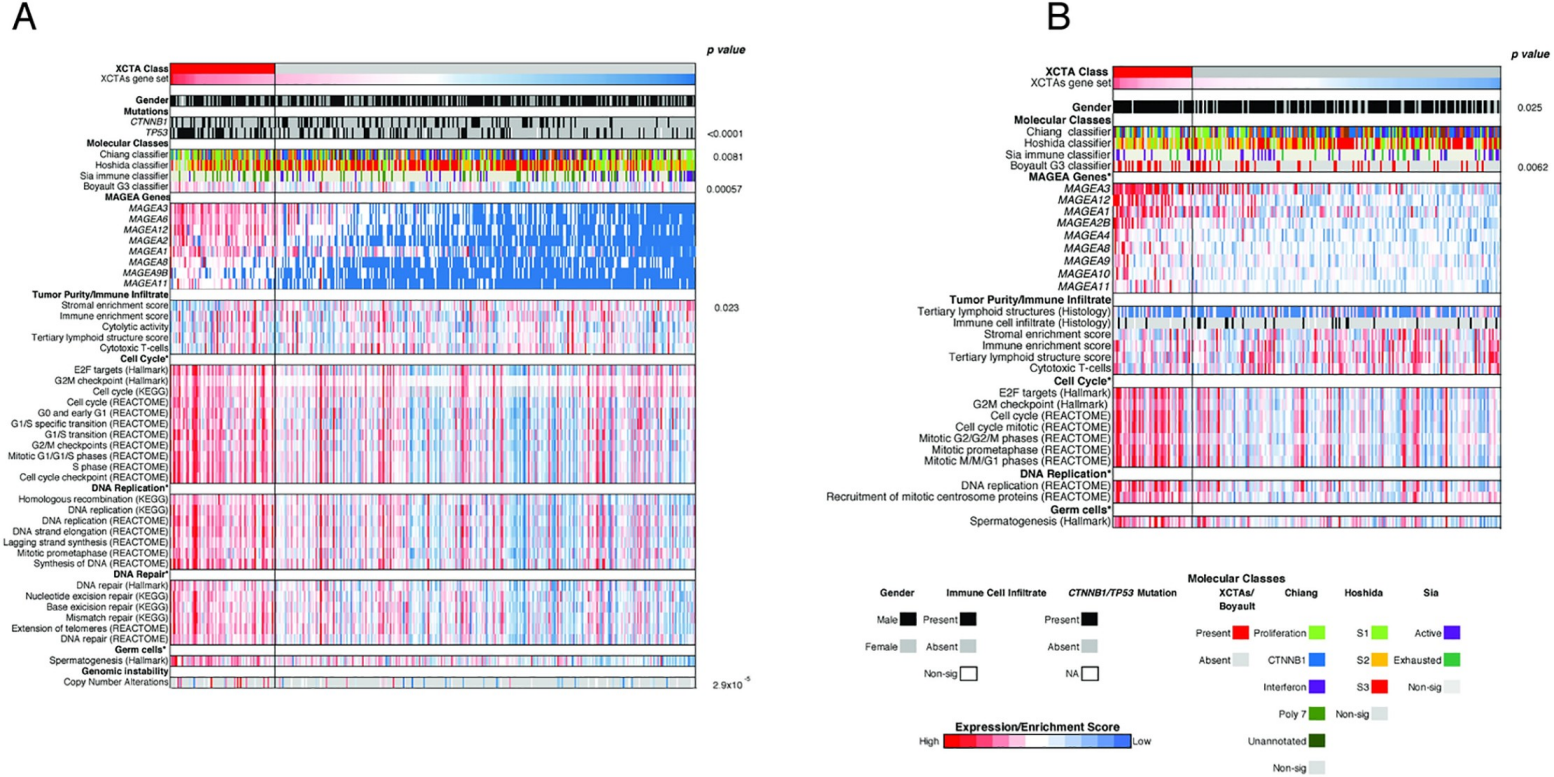
**A)** Heatmap profiling the TCGA-LIHC HCC cohort. **B)** Heatmap profiling the Heptromic cohort. In both datasets, tumors were classified according to XCTA expression levels into XCTA high (red) or low (grey) (XCTA high = top 20% expressors). All Hallmark, KEGG and Reactome gene sets shown are enriched for the XCTA high class of patients (FDR<0.05). P-values for TP53 mutations, gender, Boyault G3 classifier (Heptromic cohort) and Chiang molecular classification calculated by Pearson’s chi-squared test. P-value for Boyaults G3 classifier (TCGA-LIHC HCC cohort), stromal enrichment score and copy number alterations calculated by t-test.

To comprehensively profile tumors with high XCTA expression, we generated a XCTA score (see Materials and methods for details). We assigned tumors from two patient cohorts, TCGA-LIHC HCC (n = 361) and Heptromic (n = 228), into XCTA high or low groups based on the score (top 20% classified to the XCTA high group in each cohort) and compared differences in clinical and molecular features between the two classes. *MAGEA3* expression was a major contributor in the classification by XCTA score enrichment, as it was 2nd in the XCTA gene rank ordered list when the high/low groups were compared to each other using GSEA (**S5 Table**). Upon classifying patients into high and low expressers of XCTAs, several distinct differences between these patients are observed. First, patients in the XCTA-high class are significantly enriched in gene signatures correlated with poor clinical outcomes such as the G3 class (p = 0.0057 & 0.0062, t-test & Pearson’s chi-squared test, respectively, **Fig 2A**) [30]. Similarly, patients in the XCTA-high class in the TCGA-LIHC cohort were enriched in the Proliferation class (p = 0.0081, Pearson’s chi-squared test **Fig 2A and 2B)** [31]. There was no difference in Immune classification [32] as well as several gene sets associated with immune infiltration between the XCTA-high and XCTA-low molecular classes. This indicates that differences in expression between the two groups are not solely due to differences in tumor purity, consistent with our previous findings in the multi-regionally sampled cohort [32]. Patients in the XCTA-high class had tumors significantly enriched in gene sets involved in cell cycle, DNA replication, DNA repair, germ cells, and genomic instability (FDR< 0.05, **Fig 2A and 2B**). Last, tumors in the XCTA-high class were also significantly more likely to have *TP53* mutations, another feature associated with poor prognosis (p < 0.0001, Pearson’s chi-squared test).

**Fig 3:**
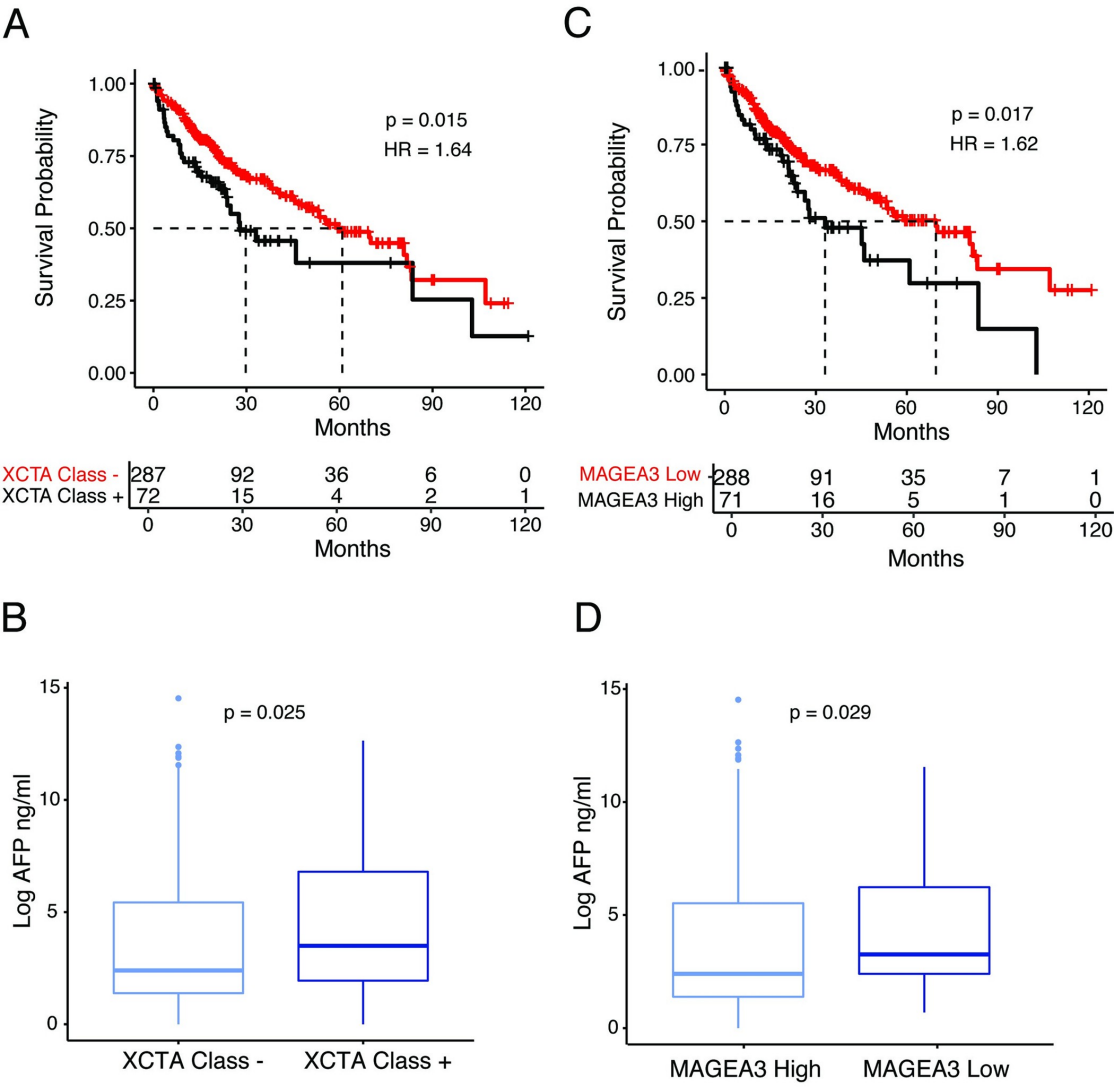
**A)** Kaplan-Meier curve showing the percentage of survival in XCTA high (+) and XCTA low (-) patients. **B)** Box plot showing the differences in AFP levels between XCTA high (+) and XCTA low (-) patients. **C)** Kaplan-Meier curve showing the percentage of survival in MAGEA3 high and MAGEA3 low patients (MAGEA3 high = top 20% expressors) **D)** Box plot showing the differences in AFP levels between MAGEA3 high and MAGEA3 low patients. All analysis was performed on data from the TCGA LIHC HCC cohort.

We also observed several associations between the XCTA-high class and clinical parameters. Patients classified as high XCTA expressors had significantly worse overall survival (HR = 1.64) in the TCGA-LIHC HCC cohort compared to low XCTA expressors and were associated with significantly higher levels of AFP, a poor prognostic factor in HCC (p = 0.015 and 0.025, respectively, **Fig 3A and 3B**). Considering the major contribution of *MAGEA3* re-expression to the XCTA enrichment signature, we further investigated the association of *MAGEA3* expression alone with clinical parameters. Indeed, high *MAGEA3* expression is also associated with poor overall survival (HR = 1.62) and higher levels of AFP in the TCGA-LIHC cohort (p = .0.017 and 0.029, respectively, **Fig 3C and 3D**). In summary, XCTA and *MAGEA3* expression correlate with clinical, histological and molecular features suggestive of aggressive HCC.

### MAGEA3 is a diversely expressed driver of proliferation in HCC

**Fig 4:**
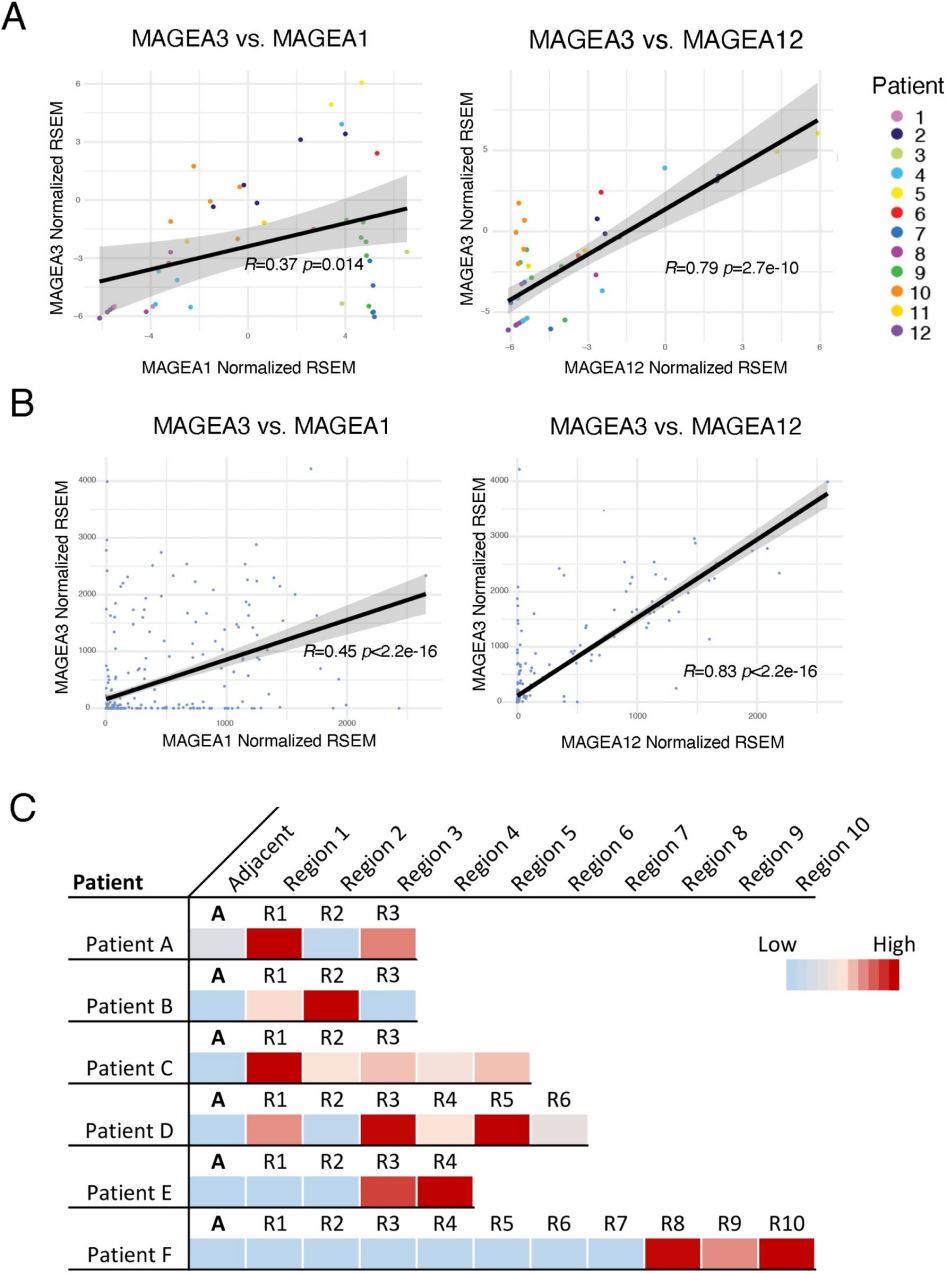
**A)** Scatter plot showing the correlation between the expression of *MAGEA3* and other close members of the MAGE-A family (*MAGEA1* and *MAGEA12*) in the multi-regionally sampled cohort. **B)** Scatter plot showing the correlation between the expression of *MAGEA3* and other close members of the MAGE-A family (*MAGEA1* and *MAGEA12*) in the TCGA-LIHC HCC cohort. **C)** Heatmap showing the heterogeneity in the expression levels by RT-PCR of *MAGEA3* in different regions of the same tumor in a secondary multi-regionally sampled cohort.

Due to the specific up-regulation of *MAGEA3* in high-grade tumors and association with worse overall survival, we decided to focus specifically on the role of *MAGEA3* in HCC. Since the MAGE-A gene family is highly conserved, we were first interested in the relationship of *MAGEA3* expression to expression of other MAGE-A genes [19]. Comparing the expression of *MAGEA3* to *MAGEA1* and *MAGEA12*, we observed a strong correlation of expression to these members of the MAGE-A family in our multi-regionally sampled cohort, as has been previously described in other cancers (R = 0.37 p = 0.014, R = 0.79 p = 2.7e-10, respectively **Fig 4A**). We further validated co-expression of the MAGE-A gene family in the TCGA-LIHC and Heptromic cohorts (**Figs 4B and S2**). To further confirm the intra-tumoral heterogeneous distribution of XCTA expression, we analyzed the expression of *MAGEA3* in a secondary multi-regionally sampled cohort by RT-PCR (n = 14, 69 samples, median 4 regions per patient). 6/14 patients (42%) expressed *MAGEA3* in our validation cohort compared to 7/12 (58%) of our initial dataset. Intra-tumoral expression differences were observed in 4/6 (67%) patients who expressed *MAGEA3* (FC>10 to at least one other region, **Fig 4C**).

**Fig 5:**
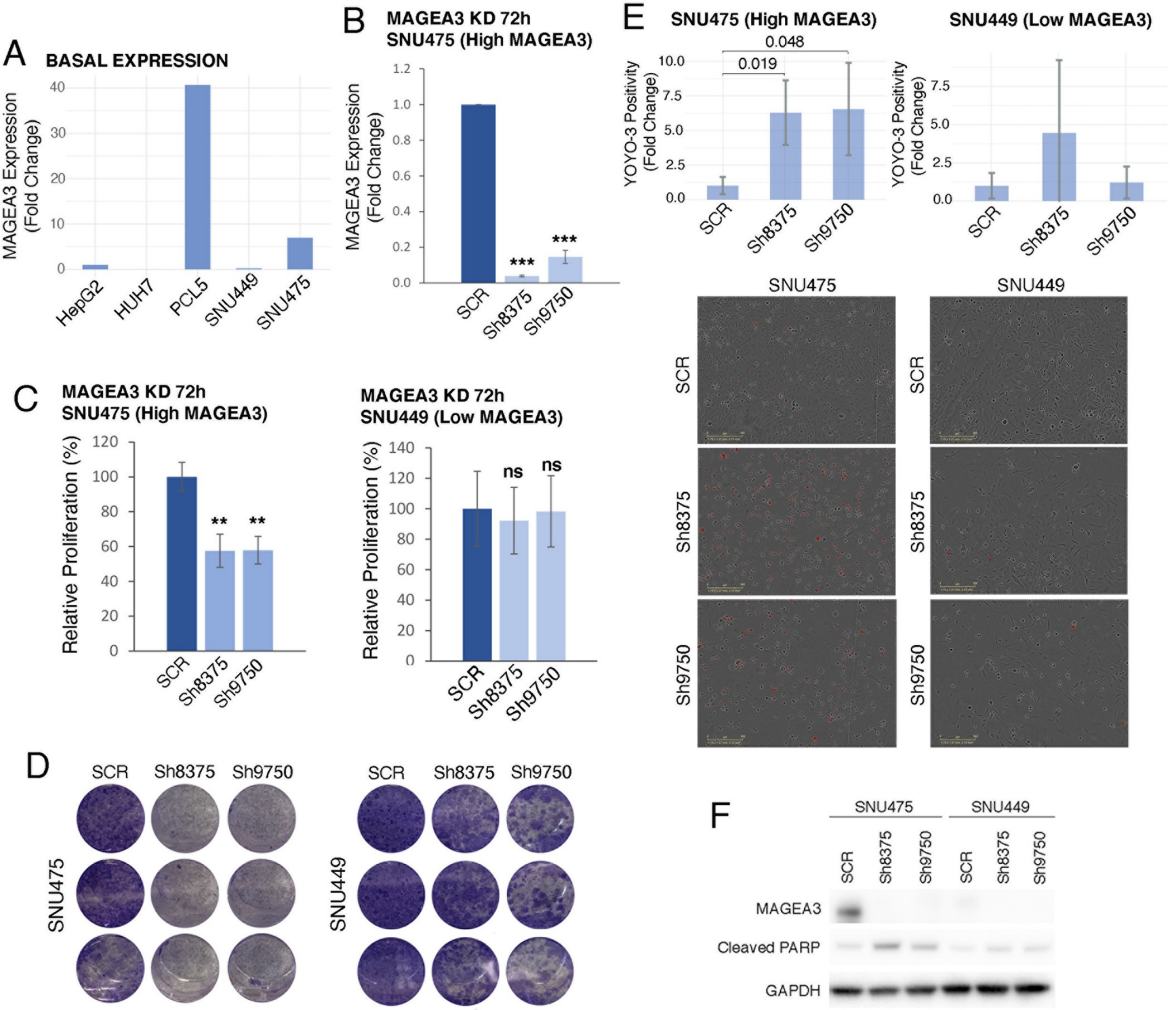
**A)** Bar graph showing the basal expression of *MAGEA3* in five different HCC human cell lines (RT-PCR, fold change compared to HEPG2). **B)** Bar graph showing *MAGEA3* expression after MAGEA3 KD with short hairpin RNAs sh8375, sh9750 or SCR control in SNU475 cell line. Fold change is relative to SCR control. **C)** Bar graph showing proliferation levels (cell viability assay) of SNU475 and SNU449 cells after MAGEA3 KD in relation to SCR control. **D)** Clonogenic assays showing the effects of MAGEA3 KD on colony formation in SNU475 and SNU449 cells. E) Bar graph showing relative quantification to SCR control of apoptotic cells (stained with YOYO-3) after 72h of MAGEA3 KD in SNU475 and SNU449 cells. Below, representative images illustrating the reduction in the number of cells after MAGEA3 KD and the increase in apoptosis (red staining). F) Western blot showing MAGEA3, cleaved PARP and GAPDH protein levels after MAGEA3 KD in SNU475 and SNU449 cell lines. *<0.05, **≤0.001, ***≤0.0001.

For a functional assessment of MAGEA3 as a driver of HCC progression, we performed short-hairpin RNA (shRNA) mediated knockdown of *MAGEA3* in 4 human HCC cell lines. Based on previously published microarray expression data and confirmation by RT-PCR, two high *MAGEA3* expressing cell lines (PLC5 and SNU475) were chosen for further experiments (**Figs 5A and S3A**). SNU449 and HEPG2 were also included for control experiments, as they express low levels of *MAGEA3* (**Figs 5A and S3A**). More than an 80% knockdown of *MAGEA3* at the RNA level was achieved with two different shRNAs (sh8375 and sh9750) in SNU475 and PLC5 compared to scramble control (p≤0.0001 **Figs 5B and S3B**). There was a significant decrease in cell proliferation of SNU475 and PLC5 after seventy two hours of *MAGEA3* down-regulation (50–60%, p≤0.001, 25–35%, p≤0.0001, **Figs 5C and S3C**). A clonogenic assay also showed a decrease in colony formation following *MAGEA3* knockdown with sh8375 and sh9750 in both cell lines (**Figs 5D and S3E**). After documenting a decrease in proliferation, we next tested for cell death by looking for an increase in apoptosis markers. Seventy-two hours after knockdown, cells staining for the apoptosis marker YOYO-3 were counted using the Incucyte S3 live cell imager. There was more than a 3-fold increase in YOYO-3 positive cells in SNU475 (p = 0.019, p = .048, respectively) and PLC5 (p = 0.05, p = 0.002, respectively) after knockdown with both shRNAs (**Figs 5E and S3D**). By western blot, cleaved PARP was increased after knockdown with both shRNAs in both cell lines (**Figs 5F and S3F**). We confirmed a decrease in MAGEA3 protein levels by Western blot after knockdown with sh8375 in both PLC5 and SNU475 but could only confirm a decrease in MAGEA3 protein after knockdown with sh9750 in the SNU475 cell line (**Figs 5F and S3F**). This may be due to the high homology of MAGE-A proteins and the promiscuity of the antibody, which has been previously documented for other MAGEA3 antibodies [22]. RNA expression of *MAGEA3* was barely detected in the cell line HEPG2 and not detected in the cell line SNU449 either before or after treatment with shRNAs or the scramble control. When treated with both shRNAs, there was no effect on proliferation in HEPG2 and SNU449 or apoptosis in SNU449, suggesting an on-target source for the effects seen in PLC5 and SNU475 (**Figs 5 and S3**). This data shows a dependence of two human HCC cell lines on MAGEA3 for maximum proliferative capacity.

**Fig 6:**
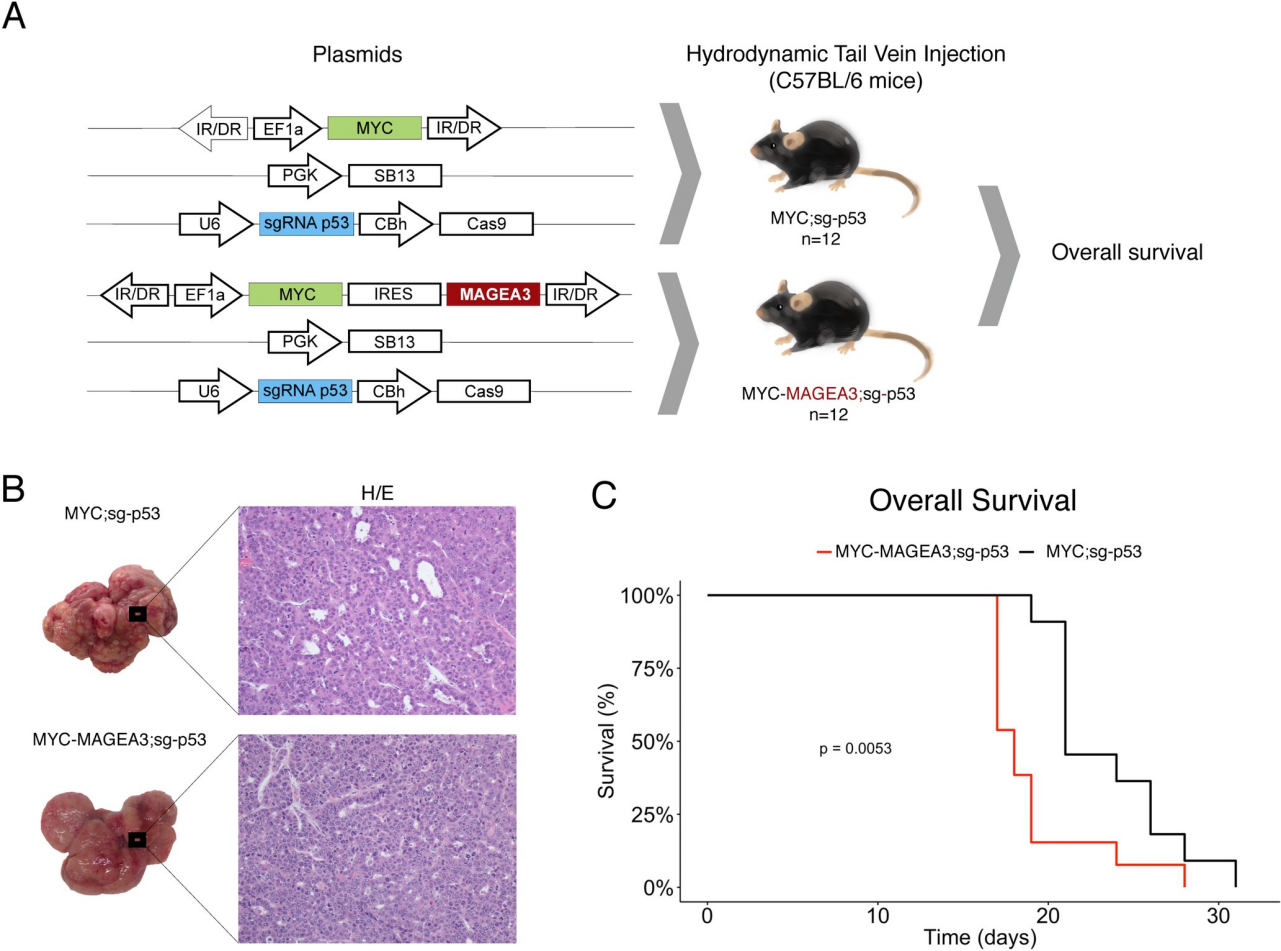
**A)** Schematic representation of the plasmids used for *in vivo* MAGEA3 overexpression in each experimental group. The experimental design includes one control group MYC;sg-p53 (n = 12) and one group overexpressing the gene of interest MYC-MAGEA3;sg-p53 (n = 12). Plasmid transfection was performed by hydrodynamic tail vein injection in C57BL/6 mice. **B)** Representative images of the macroscopic appearance of the liver tumors in both experimental groups (MYC;sg-p53 and MYC-MAGEA3;sg-p53) and its corresponding histological images (20X). **C)** Kaplan-Meier curve showing the overall survival associated with MYC;sg-p53 mice and MYC-MAGEA3;sg-p53 mice. *SB13*, *Sleepy Beauty 13 (transposon system);* CBh, *chicken beta actin short (promoter); EF1α*, *elongation factor 1 α (promoter); IRES*, *internal ribosomal entry site; IR/DR*, *inverted repeat structure; PGK*, *phosphoglycerate kinase (promoter); sg-*p53, p53 single guide RNA; *U6*, III RNA polymerase III promoter.

To test if MAGEA3 contributes to HCC progression, we utilized a well-established genetic mouse model of HCC (Myc;sg-p53 [33]). Transposon vectors for MYC overexpression (pT3- EF1a-Myc) and a single guide RNA (sgRNA) targeting TP53 (px330-sg-p53) are co-delivered with a sleeping beauty transposase plasmid (PGK-SB13) into hepatocytes of C57BL6 mice by hydrodynamic tail-vein injection (**Fig 6A**). This model reliably produces liver tumors in approximately 3–4 weeks and has all the histologic features of a human HCC. We hypothesized that if MAGEA3 plays an active role in HCC progression, the additional overexpression of MAGEA3 in this model would lead to accelerated tumor development. To test this, we developed a MAGEA3 and MYC overexpression transposon vector (pT3-EF1a-Myc-Ires-MAGEA3) to compare the rate of tumor development against the MYC transposon vector alone (**Fig 6A**). 12 mice per group were injected with either MYC;sg-p53 or MYC;MAGEA3;sg-p53. All mice from both conditions developed tumors and were histologically both dedifferentiated, as expected due to the aggressive nature documented for the baseline MYC;sg-p53 tumor model (**Fig 6B**). Due to the experimental design endpoint of this experiment being survival, the tumor burden within the livers was very high, making it impossible to count individual tumors. All mice were included in the survival study. Mice included in the MYC-MAGEA3;sg-p53 arm had significantly worse survival than mice in the MYC;sg-p53 arm (**Fig 6C**). This provides *in vivo* evidence that MAGEA3 contributes to HCC aggressiveness and progression.

### MAGEA3 depletion induces apoptosis by inhibiting Survivin

**Fig 7:**
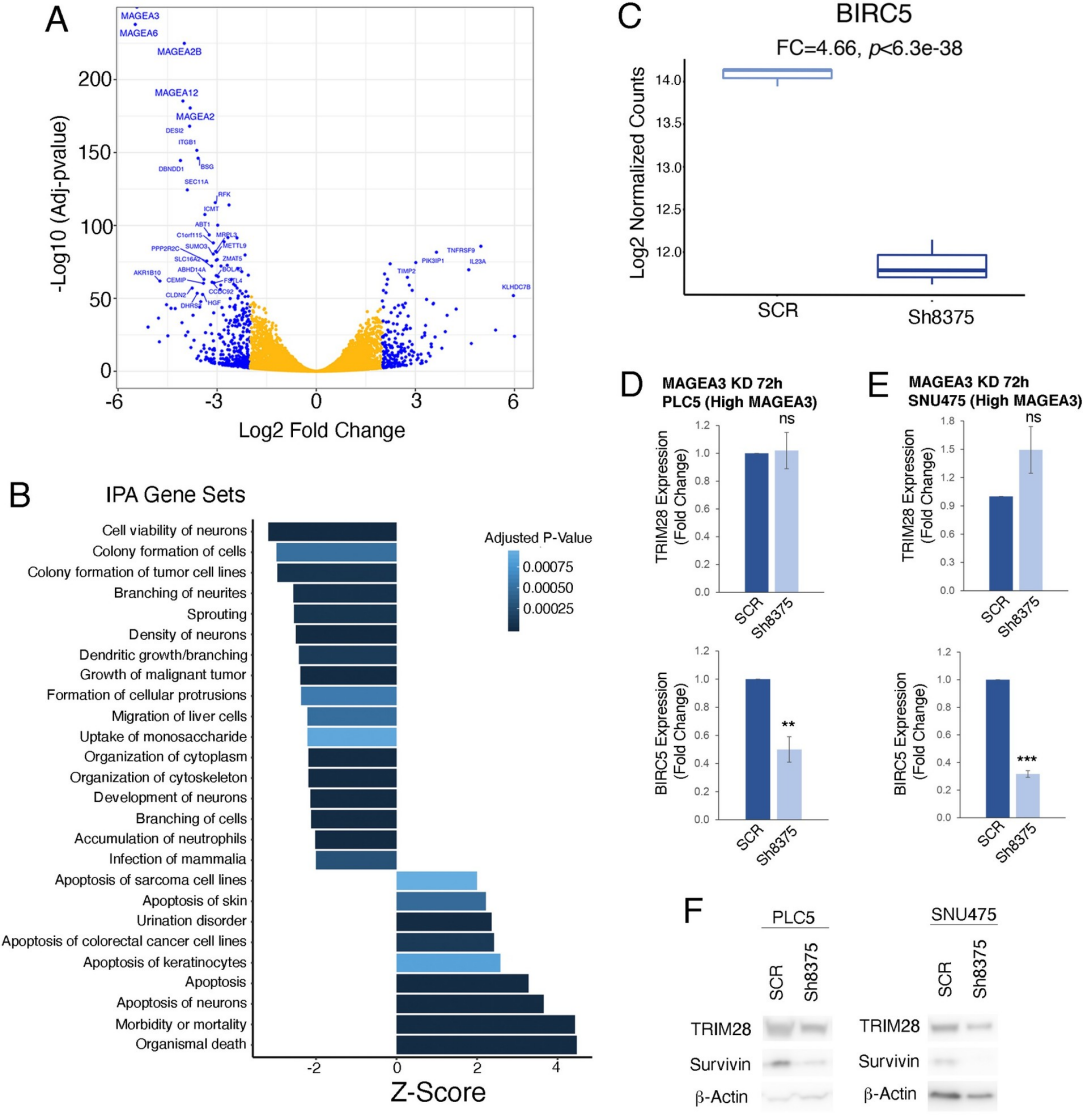
**A)** Volcano plot showing differential gene expression between SCR treated and MAGEA3 knockdown after 72 hours in the PLC5 cell line. **B)** Signaling pathways regulated after 72h of MAGEA3 KD in the PLC5 cell line. **C)** Box plot showing the differences in *BIRC5* (Survivin) expression between SCR control and MAGEA3 KD in the PLC5 cell line. **D)** Bar graph showing *TRIM28* and *BIRC5* (Survivin) expression after MAGEA3 KD with short hairpin RNAs sh8375 or SCR control in the PLC5 cell line**. E**) Bar graph showing *TRIM28* and *BIRC5* (Survivin) expression after MAGEA3 KD with short hairpin RNAs sh8375 or SCR control in the SNU475 cell line. **F)** Western blot showing TRIM289, Aurvivin and B-Actin protein levels after MAGEA3 KD in PLC5 and SNU475 cell lines. *<0.05, **≤0.001, ***≤0.0001.

To better understand the mechanism by which MAGEA3 contributes to HCC progression, we conducted RNA sequencing in the PLC5 HCC cell line after 72 hours of treatment with scramble or sh8375 in triplicate. First, we performed differential expression analysis between scramble control and MAGEA3 knockdown conditions. Confirming that our knockdown worked efficiently, *MAGEA3* was the most significantly down-regulated gene (**Fig 7A and S6 Table**). In addition to *MAGEA3*, several other MAGE-A genes were also down-regulated, including *MAGEA2B*, *MAGEA12*, and *MAGEA2*. This could be due to the direct targeting of these genes by the shRNA because of the high homology between the MAGE-A family of genes.

To better understand the cellular signaling disruptions caused by down-regulation of MAGEA3, we performed gene set enrichment analysis (GSEA) with Ingenuity Pathway Analysis (IPA, **S7 Table**). Significant positively enriched gene sets were associated with apoptosis, while significant negatively enriched gene sets were associated with cell viability, colony formation and growth (**Fig 7B**). Due to the strong signal of cell viability and apoptosis, we became interested in the role *MAGEA3* plays in these processes. *MAGEA3* has previously been described to prevent apoptosis through the upregulation of *BIRC5* (Survivin), a member of the inhibitor of apoptosis family, in multiple myeloma [23]. Indeed, we also find a significant decrease in *BIRC5*, suggesting that this may also be a mechanism by which *MAGEA3* protects against apoptosis in HCC (p<6.3e-38, FC = 4.66, **Fig 7C and S6 Table**). We confirmed a decrease in *BIRC5* (Survivin) after MAGEA3 depletion by sh8375 at both the RNA and protein levels in both PLC5 and SNU475 cell lines (**Fig 7D, 7E and 7F**). We also investigated the impact of MAGEA3 depletion on the well-established binding partner of MAGEA3, a ubiquitin ligase called TRIM28. We see no significant changes in *TRIM28* RNA levels after MAGEA3 knockdown either by RNA-seq or RT-PCR, while we see a slight reduction of TRIM28 protein levels by Western blot (**Fig 7D, 7E and 7F and S6 Table**). Taken together, inhibition of MAGEA3 led to a significant increase in apoptosis via loss of Survivin, further suggesting the role of MAGEA3 in tumor progression.

## Discussion

Here we present a comprehensive analysis of XCTA gene family expression in HCC and describe the active role of MAGEA3 in promoting tumor progression. We utilized our unique multi-regionally sampled cohort to identify genes that are activated as individual tumors progress from low to high tumor grades. We hypothesized that these genes may play an active role in driving HCC into a more aggressive state, providing novel vulnerabilities that can be exploited therapeutically. To identify these genes, we compared the differentially expressed genes in tumors with regional variation in histological grade (i.e., well and poorly differentiated). By genetically characterizing different histological aggressive areas within a single patient, we eliminate inter-patient variation that influences comparisons between groups of patients. Thus, by examining intra-tumor heterogeneity the most likely candidates of tumor progression can be identified on a per patient basis, which is not possible with large single biopsy cohorts such as the TCGA.

In addition to our unique per-patient analysis approach, we believe transcriptional profiling, as opposed to identifying mutations, is an underutilized method to determine novel drivers of tumor progression. The most common mutations in HCC affect *TERT* promoter, *TP53* and *CTNNB1*, all of which are currently undruggable. New approaches such as ours facilitate the discovery of non-mutated therapeutic targets and helps fulfill a clear unmet need. Epigenetically regulated oncogenes have been described in HCC. For example, IGF2 is regulated by promoter methylation and overexpression leads to enhanced HCC proliferation in several experimental models [34]. Acknowledging the limitation regarding mutations, we focused directly on altered gene expression within tumors that may be regulated by alternative methods other than DNA alterations. While our study was initially designed to assess individual drivers of progression, to our surprise one group of genes was frequently observed with significantly higher expression in poorly differentiated regions in our patients. This group was the XCTA family. Initially discovered in melanoma as tumor specific antigens, XCTAs have been investigated as key instigators of cytotoxic immune responses [7]. Interestingly, we did not see evidence of any correlation with TILs and CTA expression (previously published [27]). To evaluate the proportion of HCC patients that may similarly upregulate XCTAs upon development of poorly differentiated tumors, we created a XCTA score to classify patients in single biopsy cohorts as high or low expressors. Based on these evaluations, the profile of patients who have high expression of XCTAs, specifically *MAGEA3*, encompasses multiple markers of poor prognosis. This prompted us to further investigate if MAGEA3 is merely a new biomarker of poor prognosis or if it plays an active oncogenic role in HCC progression.

While the MAGE-A family of XCTAs have mainly been investigated for their utility in immunotherapies, studies have found that they have oncogenic properties in several cancers, including HCC [35]. Most recently MAGEA3 was functionally characterized as having a pro-tumor role in experimental models of pancreatic cancer and HCC [36,37]. MAGEA3 mediates increased proliferation and chemoresistance against cytotoxic agents in pancreatic and HCC (HEPG2 & HUH7) cell lines. Additionally, MAGE-A proteins can inhibit autophagy and promote glycolysis in cells under metabolic stress [9,38]. To establish a mechanism by which MAGEA3 drives tumor progression in HCC, we performed RNA sequencing on the PLC5 cell line after knockdown with sh8375. A strong knockdown of MAGEA3 was confirmed, along with a down-regulation of several other MAGE-A family genes that share a high homology with MAGEA3. The most prominently induced gene sets were related to apoptosis. This was consistent with our *in vitro* apoptosis assay results and previous reports of MAGEA3 as a regulator of apoptosis in multiple myeloma through both TP53 dependent and independent mechanisms [23]. Independently of TP53, MAGEA3 was shown to stabilize levels of Survivin, an anti-apoptosis regulator. Survivin prevents apoptosis through the inhibition of caspases. When we checked the levels of *BIRC5* (Survivin), we saw a significant decrease after MAGEA3 knockdown at both the RNA and protein levels. These data in conjunction with the fact that PLC5 and SNU475 used for our *in vitro* studies are both TP53 mutants suggests that MAGEA3 may regulate apoptosis in a TP53-independent manner through the stabilization of Survivin similar to what has been described in multiple myeloma [23]. Survivin stabilization prevents apoptosis that would otherwise be triggered by the increased proliferative capacity acquired by HCC cells.

We acknowledge that while these data are a promising first step toward confirming the oncogenic nature of MAGEA3, our study has some limitations. First, the multi-regionally sampled cohort for which XCTA ITH was established is relatively small (26 patients). Additionally, since the TCGA-LIHC and Heptromic cohorts are composed of single biopsies samples, we cannot observe the intra-tumor heterogeneity of *MAGEA3* expression in these patients. Thus, we could be underestimating how many patients have expression of *MAGEA3*, which according to our data are more likely to have a poor prognosis. Also, due to the high similarity between MAGEA3 and other members of the MAGE-A family, we cannot rule out functional redundancy between MAGEA3 and other genes of the family. In terms of oncogenic mechanisms related to MAGEA3 overexpression, previous studies mainly converge on the process of apoptosis evasion, either through regulation of ubiquitin ligases such as TRIM28, which target TP53 for proteasomal degradation, or stabilization of Survivin [39]. Aside from regulating apoptosis, there is also evidence in HCC that MAGEA3 regulation of TRIM28 leads to the degradation of FBP1, a key regulator of the Warburg effect [35]. In our study, we see no significant changes in TRIM28 RNA or protein levels after MAGEA3 knockdown, although this does not disqualify that MAGEA3 may regulate TRIM28 function through direct protein-protein interactions as observed in the degradation of FBP1. Aside from FBP1, we do not know other downstream targets of TRIM28-MAGEA3 in HCC. Discovering novel targets is an important area of further study in the field. The mechanism of MAGEA3 de-regulation is not well understood, but may be due to DNA hypomethylation of promoter regions. We provide evidence that this may be the case in HCC, as we see strong correlations in expression of various MAGE-A family genes with *MAGEA3*, that are found in close genomic proximity to each other, which may be due to hypomethylation in this region of the X chromosome. Importantly, different levels of promoter methylation across individual tumors may also be the cause of variation in *MAGEA3* expression we observe in our multi-regionally sampled dataset. MAGEA3 therapeutic inhibition may be achieved by direct targeting of MAGEA3 or targeting upstream regulatory factors that repress its expression. Interestingly, MAGEA3 is targeted by noncoding RNAs, including mir-31-5p, by *in vitro* experiments using HCC cell lines [37]. When mir-31-5p was experimentally depleted, *MAGEA3* expression increased, leading to higher levels of proliferation and invasion. While not presented here, future comprehensive profiling of upstream XCTA regulation including promoter methylation and miRNA binding prediction would greatly increase the feasibility of therapeutic development to disrupt XCTAs, and more specifically MAGEA3, function.

In conclusion, we used a unique multi-regionally sampled cohort expression dataset to identify novel drivers of tumor progression beyond driver genes with DNA alterations. This study adds data to a growing field of evidence in support of MAGEA3 as a driver of tumor progression and a potential novel therapeutic target in human cancer.

## Materials and methods

### Ethics statement

All patients of the multi-regionally sampled cohort were enrolled at the Icahn School of Medicine at Mount Sinai (ISMMS) and provided informed consent for tissue biobanking. Formal consent was obtained both verbally and in writing. and samples were provided by the ISMMS Tissue Biorepository. This study was approved by the IRB (IRB# HS-14-01011).

### Human samples and histological evaluation

All patients of the multi-regionally sampled cohort had HCC as per EASL guidelines [40] and were treatment-naïve prior to resection. Frozen tissue samples were collected allowing for at least one cm of distance between each other. For morphological analysis, sections were cut (5μm thick), stained with hematoxylin and eosin (H&E) and evaluated by an expert liver pathologist. The histological features evaluated included tumor grade by the Edmondson criteria (i.e., well, moderately and poorly differentiated), a semi-quantitative evaluation of immune cell infiltrate and steatosis (absent, mild, moderate and severe), and enumeration of mitotic figures per high-power field. Degree of fibrosis in the adjacent non-tumoral liver was assessed using the METAVIR scoring system [41]. Histological evaluation and gene expression data has been previously reported [27].

### Regional expression variance

To account for regional gene expression changes, we carried out statistical tests for differential expression across all combinations of regions within a given patient by testing the null hypothesis that the logarithmic fold change (LFC) between regions for a given gene’s expression is zero. For patients with three or more regional samples, we compared all unique regional combinations building from 2x1 comparisons. In order to facilitate gene ranking, stable effect size estimation, and variance sharing across genes among samples we used DESeq2 [42] to model the dependence of the dispersion of the count data on the average expression strength over all of the samples in the comparison. Since all comparisons were between samples on the same genetic background, tissue type, and sequencing run, we simply imposed a more stringent false discovery rate (FDR, Benjamini and Hochberg method) of 1% to account for the inherent lack of power of these statistical tests. Gene expression correlation between regions was tested using Spearman’s rank correlation. Figures were constructed using normalized counts.

### TCGA-LIHC HCC and Heptromic Cohorts

The TCGA data was downloaded from the National Cancer Institute’s GDC Data Portal (https://portal.gdc.cancer.gov/) for HCC patients. Matched clinical data was downloaded from the cBioPortal (http://www.cbioportal.org/) [43]. The Heptromic Cohort expression data is deposited at the Gene Expression Omnibus (GSE63898) [44]. Sample collection is described extensively in the cited publications for each of these cohorts. Briefly, all samples from the TCGA-LIHC and Heptromic cohorts were fresh-frozen. Patients also had no prior treatment to resection. Samples that underwent sequencing from both cohorts were required to pass multiple quality control parameters. Specifically, in the TCGA-LIHC cohort, samples were required to include ≥ 60% tumor nuclei and ≤ 20% necrosis. Figures from TCGA-LIHC HCC data were constructed from RSEM normalized counts. Figures from the Heptromic Cohort were constructed from log normalized intensity expression values.

### RNA sequencing of tissue samples and cell lines

RNA-seq of the multi-regionally sample cohort has previously been described [27]. RNA-seq of cell lines was conducted on poly-A enriched RNA, 100 bp paired end reads using an Illumina HiSeq2500 instrument. Libraries were constructed using the TruSeq RNA Library Prep Kit v2. Raw sequencing reads were mapped to the GRCh38 reference genome (USCS) using STAR (2.4.2g1) [45]. Aligned reads were mapped to GRCh38 genetic features using featureCounts from the subRead package [46] with default settings. RNA sequencing data of the cell lines has been deposited at ArrayExpress (E-MTAB-8860).

### Gene set enrichment analysis and XCTA score calculation

Single sample gene set enrichment analysis (ssGSEA) [29,47] was used to determine enrichment scores for the Hallmark, KEGG and REACTOME gene sets (www.gsea-msigdb.org) for samples of the TCGA-LIHC HCC and Heptromic Cohorts using GenePattern. FDR values were obtained by comparing the tails of the observed and null distributions for the enrichment scores. Gene signatures composed of testis-expressed CTAs for the CTA score and X chromosome located CTAs for the XCTA score were self-curated using the CTDatabase (http://www.cta.lncc.br/, **S3 Table**). Enrichment scores for each sample were generated using ssGSEA [47]. The CTA gene signature was used to generate scores in the Multi-regionally sampled cohort and the XCTA gene signature was used to generate scores in the TCGA-LIHC HCC and Heptromic Cohorts [48]. In order to identify which genes’ expression had the highest influence on the ssGSEA scores, we compared patients with the 20% highest XCTA scores to the patients with the lowest 80% XCTA scores using group GSEA. GSEA uses a leading edge analysis, which ranks genes and calculates a running sum statistic. Enrichment scores are the value at which the statistic is at the maximum deviation from zero. We used the rank and rank metric score from the output of this analysis to interpret which genes are most important to distinguish XCTA-high from XTA-low patients. Ranked scores are included in **S5 Table**. For the RNA sequencing of the *MAGEA3* knockdown cell lines, we used the Ingenuity Pathway Analysis software (QIAGEN Inc., https://www.qiagenbioinformatics.com/products/ingenuitypathway-analysis) to calculate enriched gene sets. Genes were ranked by differential expression between wild type and knockdown samples. GSEA analysis was performed using GenePattern (https://www.genepattern.org/).

### Human cell lines and lentiviral shRNA assay

PLC5 and HepG2 cells were maintained in DMEM Dulbecco medium with 10% FBS, 1% L-glutamine, and 1% penicillin-streptomycin. SNU449 and SNU475 were maintained in RPMI medium with 10% FBS, 1% L-glutamine, and 1% penicillin-streptomycin. PLC5 and SNU475 were confirmed as mycoplasma free using the PCR-based Venor GeM Mycoplasma Detection Kit (Sigma-Aldrich). Cell line authentication of PLC5 and SNU475 was confirmed using short tandem repeats DNA sequencing performed by a cell line sequencing service (Genetica). PLC5, HepG2, SNU475 and SNU449 cells were infected in the presence of polybrene (8μg/ml) with lentiviruses expressing either an empty vector (pLKO.1) or a shRNA against MAGEA3 (pLKO.1) from Sigma-Aldrich: shMAGEA3 #9750: TRCN00000129750; shMAGEA3 #8375: TRCN00000128375. Cells were collected 72 hours after infection and used for RNA extraction and western blotting as described below. Lentivirus was diluted either 1:10 for proliferation and apoptosis assays or 1:2 for the RNA sequencing experiment.

### RT-PCR

RNA from cell lines was extracted using the RNeasy mini kit (Qiagen). cDNA was made from 1μg of total RNA using EcoDry Premix (Clontech). The expression of MAGEA3, TRIM28, BIRC5 and 18S was determined using real-time PCR. Each cDNA sample was amplified using Taqman assays (Thermo Fisher Scientific). The taqman probes used are as follows; MAGEA3: Hs00366532_m1, 18S: Hs99999901_s1, TRIM28: Hs00232212_m1, BIRC5: Hs00153353_m1. Briefly, the reaction conditions consisted of 5 μl of diluted cDNA and 0.5 μl of taqman probe in a final volume of 10 μl TaqMan Gene Expression Master Mix (2X) (Thermo Fisher Scientific). After polymerase activation at 95°C for 10 minutes, each of 40 cycles consisted of denaturation at 95°C for 15s and annealing at 60.0°C for 60s. 18S was used as an endogenous control to normalize each sample.

### Western blot

For immunoblot analysis, RIPA buffer supplemented with protease and phosphatase inhibitors (Invitrogen) was used to lyse treated cells. The supernatant was collected by centrifugation for 20 minutes at 12,000 (g). Protein lysates were separated using SDS-PAGE gels (10%). The gels were transferred to a PVDF membrane for 2 hours at 60V (4°C). The membranes were probed using the following primary antibodies overnight at 4°C; GAPDH (clone 6C5, dilution 1:1,000) from EMD Millipore, MAGEA3 (clone 1A10, dilution 1:1,000) from Origene, TRIM28 from Abcam (polyclonal, dilution 1:1000), β-Actin from cell signaling (clone 13E5, dilution 1:1000), Survivin from cell signaling (clone 6E4, dilution 1;1000), and Cleaved PARP (ASP214, #9541, dilution 1:1,000) from Cell Signaling. After washing with PBST, membranes were incubated with the following secondary antibodies diluted in PBST with 5% BSA; anti-mouse from Cell Signaling (dilution 1:5,000) and anti-rabbit from Cell Signaling (dilution 1:5,000). Membranes were visualized with Amersham ECL prime western blotting detection reagent (GE Healthcare, RPN2236) on an Amersham imager 600 system.

### Cell viability and apoptosis assays

The MTS assay was used to measure cell viability. Briefly, 2,000–4,000 cells were seeded in 96 well plates and infected with shRNAs. 72 hours after infection, cell proliferation was assayed by adding 20μl of CellTiter 96 AQueous One Solution Reagent (Promega) to each well containing 100μl DMEM or RPMI media. Formazan formation was quantified by measuring the absorbance at wavelength 490 (nM) and normalized to wavelength 620 (nM). All measurements were made in triplicate (technical replicates). The experiment was performed in at least 3 independent experiments (biological replicates) in each cell line.

Apoptosis measurements were made using the Incucyte S3 live cell imager. Briefly, 2,000–4,000 cells were seeded in 96 well plates and infected with shRNAs. 24 hours after infection, fresh media supplemented with YOYO-3 iodide 1mM solution in DMSO (1:5,000 dilution in media) was added to the cells and the plates were placed in the Incucyte S3 live cell imager. After 48 hours, phase contrast and fluorescent images were taken of each well. The Incucyte S3 live cell imager software quantified the number of YOYO-3 positive cells (Excitation Wavelength 612 nM, Invitrogen cat. number Y3606). All measurements were made in triplicate (technical replicates). The experiment was performed by 3 independent experiments in each cell line (biological replicates).

### Vector construction

The pT3-EF1a-Myc vector was a kind gift of Xin Chen (UCSF). This vector was digested with PmeI and then InFusion HD cloning (Takara, USA) was performed to insert the Ires and MAGEA3 fragments to generate the pT3-EF1a-Myc-Ires-MAGEA3 vector. For the permanent expression of the genes of interest within mouse hepatocytes, a vector containing the sleeping beauty transposase (PGK-SB13, Addgene plasmid #20207) was used in conjunction with the pT3-EF1a-Myc-Ires-MAGEA3 vector. For TP53 deletion, the CRISPR-Cas9 system was used [33]. Briefly, px330 vector was digested with BbsI and ligated with annealed oligonucleotides. pX330-U6-Chimeric_BB-CBh-hSpCas9 was a gift from Fen Zhang (Addgene plasmid #42230). All constructs were verified by nucleotide sequencing and restriction enzyme digestion.

### *In vivo* experiments

All mouse experiments were approved by the Icahn School of Medicine at Mount Sinai (ISMMS) Animal Care and Use Committee (protocol no. IACUC-2014-0229). Mice were maintained under specific pathogen-free conditions and food and water were provided ad libitum. All animals were examined prior to the initiation of studies to ensure that they were healthy and acclimated to the laboratory environment.

For the hydrodynamic tail-vein injections, a sterile 0.9% NaCl solution/plasmid mix was prepared containing 12.1 μg DNA pT3-EF1a-Myc or pT3-EF1a-Myc-Ires-MAGEA3 plus 3 μg PGK-SB13 and 10 μg of px330-p53 per 2 ml of saline. 5-7-week-old male C57BL6 (Envigo laboratories) mice were injected with the 0.9% NaCl solution/plasmid mix into the lateral tail vein with a total volume corresponding to 10% of body weight in 5–7 seconds. A total of 11–12 mice per group were successfully injected.

### Data analyses

For *MAGEA3* expression comparisons, t-test, Pearson’s Chi-Squared test, one-way ANOVA and Spearman’s rank correlation tests were used to determine p values. Kaplan-Meier survival analysis for the TCGA-LIHC cohort was performed using R with the packages Survival and Survminer. The patients with XCTA enrichment scores or *MAGEA3* expression in the top 20% were considered as the high groups. For each boxplot, the center line represents the median. Upper and lower limits of each box represent the 75th and 25th percentiles, respectively. The whiskers represent the lowest data point still within 1.5 × box size of the lower quartile and the highest data point still within 1.5 × box size of the upper quartile. All error bars represent standard deviation.

## Supporting information

S1 Fig Representative histology of well, moderately and poorly differentiated cells found at different tumor regions of patient 4. (TIF)

S2 Fig Scatter plot showing the correlation between the expression of *MAGEA3* and other close members of the MAGE-A family (*MAGEA1*, *MAGEA2B* and *MAGEA12*) in the Heptromic cohort. (TIF)

S3 Fig A) Bar graph showing the basal expression of MAGEA3 in five different HCC human cell lines (microarray). B) Bar graph showing MAGEA3 expression after MAGEA3 KD with short hairpins sh8375, sh9750 or SCR as a control in the PLC5 cell line. C) Box plot showing proliferation levels (cell viability assay) of PLC5 and HEPG2 cells after MAGEA3 KDs in relation to SCR control D) Bar graph showing relative quantification of apoptotic cells (stained with YOYO-3) after 72h of MAGEA3 KD in the PLC5 cell line. To the right, representative images of the PLC5 cell line after 72h of MAGEA3 KD. Red staining represents apoptosis. E) Clonogenic assays showing the effects of MAGEA3 KD on colony formation in the PLC5 cell line. F) Western blot showing MAGEA3, cleaved PARP, and GAPDH protein levels after MAGEA3 KD in the PLC5 cell line. *<0.05, **≤0.001, ***≤0.0001.
(TIF)

S1 Table Differential Gene Expression in Patient 4; H4.c vs all other regions. (XLSX)

S2 Table Differential Gene Expression in Patient 2; 2.a vs all other regions. (XLSX)

S3 Table X chromosome located Cancer Testis Antigens. (XLSX)

S4 Table CTA enrichment score in multi-regionally sampled cohort. (XLSX)

S5 Table XCTA rank metric score in TCGA samples. (XLSX)

S6 Table Differentially expressed genes between sh8375 treated PLC5 cells and SCR PLC5 cells. (XLSX)

S7 Table Ingenuity Pathway Analysis results between sh8375 treated PLC5 cells and SCR treated PLC5 cells. (XLSX)
