# Supplementary figures and images for: Transcriptomic characterization of cancer-testis antigens identifies MAGEA3 as a driver of tumor progression in hepatocellular carcinoma
Source: PLoS Genet. 2021 Jun 24;17(6):e1009589. doi: 10.1371/journal.pgen.1009589 (PMC8224860; doi:10.1371/journal.pgen.1009589)

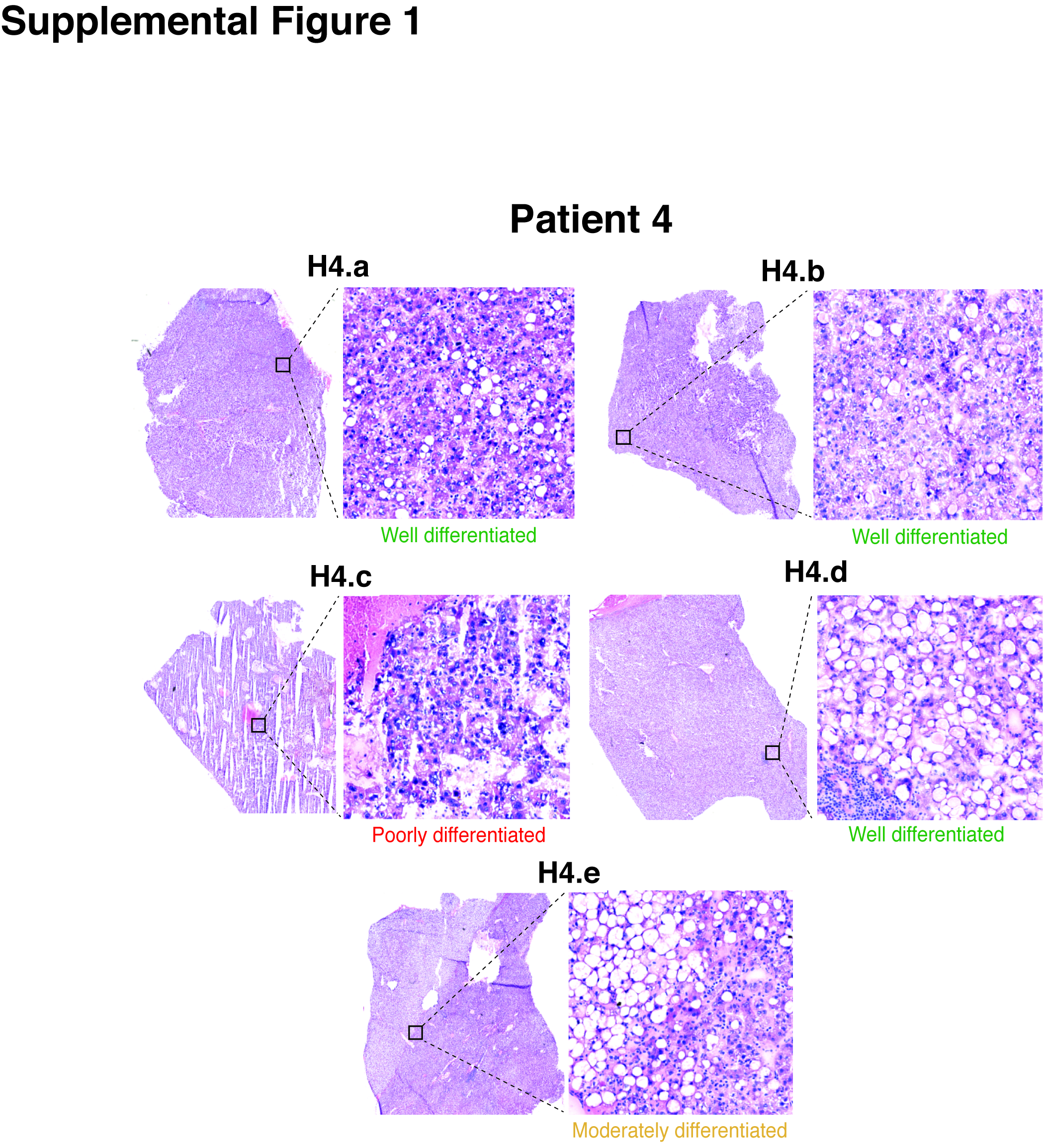

Supplement: S1 Fig — (TIF) [file pgen.1009589.s001.tif]

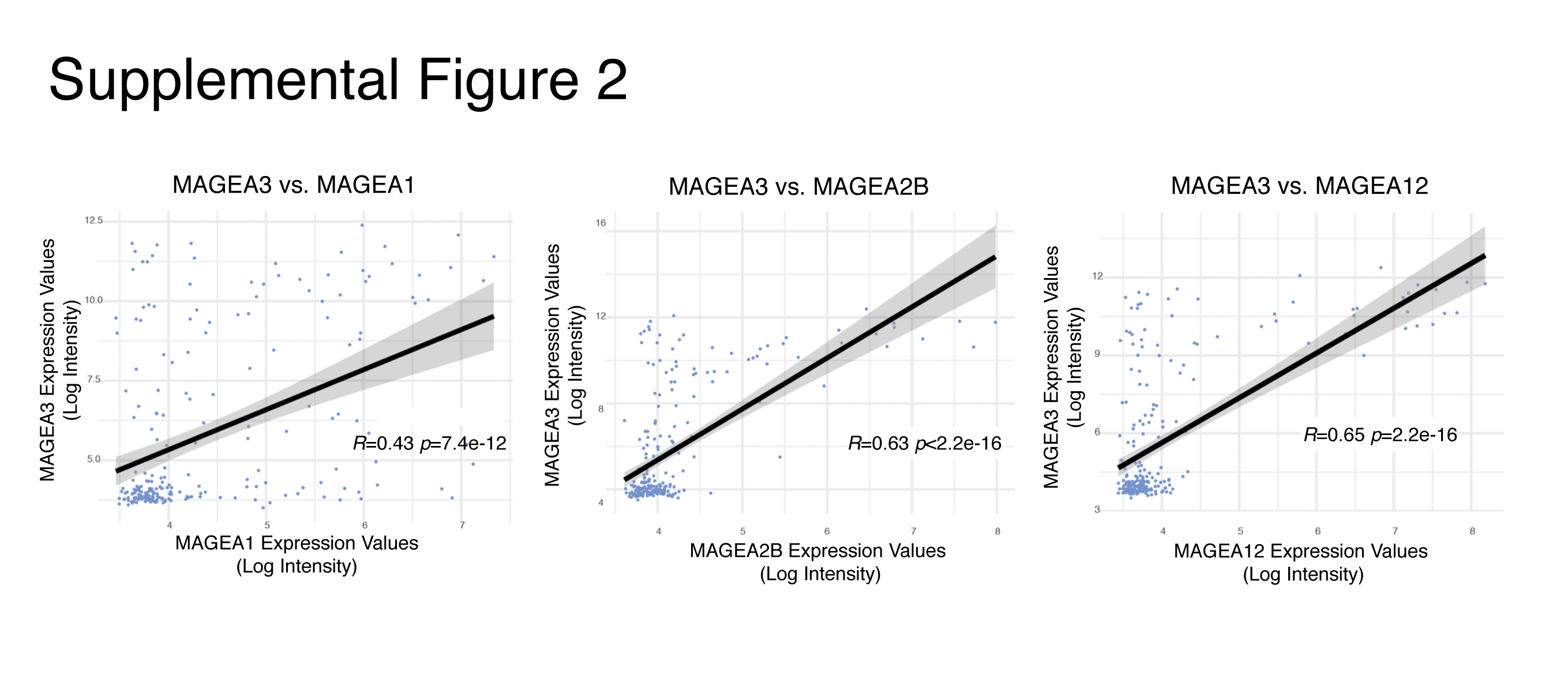

Supplement: S2 Fig — (TIF) [file pgen.1009589.s002.tif]

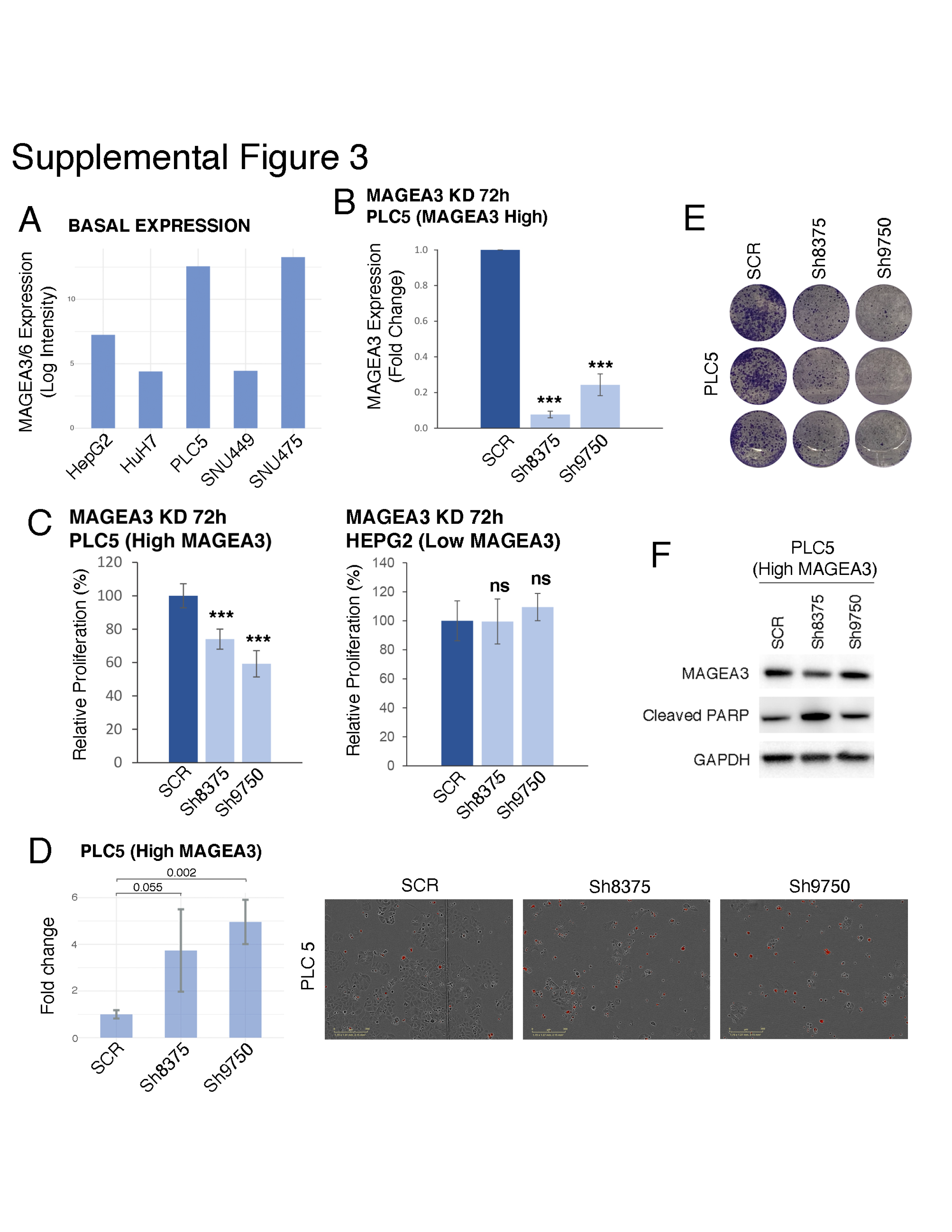

Supplement: S3 Fig — A) Bar graph showing the basal expression of MAGEA3 in five different HCC human cell lines (microarray). B) Bar graph showing MAGEA3 expression after MAGEA3 KD with short hairpins sh8375, sh9750 or SCR as a control in the PLC5 cell line. C) Box plot showing proliferation levels (cell viability assay) of PLC5 and HEPG2 cells after MAGEA3 KDs in relation to SCR control D) Bar graph showing relative quantification of apoptotic cells (stained with YOYO-3) after 72h of MAGEA3 KD in the PLC5 cell line. To the right, representative images of the PLC5 cell line after 72h of MAGEA3 KD. Red staining represents apoptosis. E) Clonogenic assays showing the effects of MAGEA3 KD on colony formation in the PLC5 cell line. F) Western blot showing MAGEA3, cleaved PARP, and GAPDH protein levels after MAGEA3 KD in the PLC5 cell line. *<0.05, **≤0.001, ***≤0.0001. (TIF) [file pgen.1009589.s003.tif]
